# Effect of Sun exposure-induced ferroptosis mechanisms on pathology and potential biological processes of primary melanoma by microarray data analysis

**DOI:** 10.3389/fgene.2022.998792

**Published:** 2022-09-26

**Authors:** Yakun Gao, Qiang Hou, Rong Guo, Jianghui Ying, Jiachao Xiong, Hua Jiang

**Affiliations:** Department of Plastic Surgery, Shanghai East Hospital, Tongji University School of Medicine, Shanghai, China

**Keywords:** Sun exposure, ferroptosis, melanoma, UV, immunotherapy targets

## Abstract

**Objectives:** Sunlight exposure is an important environmental factor in the pathogenesis of skin cutaneous melanoma (SKCM). Ultraviolet (UV) from sunlight can cause excessive intracellular production of reactive oxygen species (ROS), resulting in damage from oxidative stress to cells. As a major iron-rich and ROS-producing organelle, mitochondria are considered as an important place for cell ferroptosis. Thus, the pathology and potential biological process of UV exposure-induced ferroptosis in the development of SKCM has aroused our strong interest.

**Methods:** Gene expression profile datasets of melanoma cell line datasets (GSE31909) and UV-irradiated mitochondria dataset (GSE3632) were downloaded from the Gene Expression Omnibus (GEO) database, and ferroptosis-related genes were obtained from the FerrDb v2 database. After identifying the common differentially expressed genes (DEGs), comprehensive analyzes were performed, including functional annotation, protein-protein interaction (PPI) network construction, hub gene identification, and gene and tissue protein expression levels, survival analysis, and immune cell infiltration analysis.

**Results:** A total of 14 common DEGs was identified for subsequent analyses. Seven DEGs, including PSMB4, CRELD2, CDKN2A, TIMP1, NDRG1, ATF3 and JUND, have consistent performance in mRNA and protein expression in normal skin and SKCM tissues can be regarded as a good biomarker with SKCM diagnostic effectiveness. Functional enrichment analysis results indicate that HIF-1 signaling pathway and angiogenesis involved in the pathogenesis and development of SKCM. Induction of ferroptosis in tumor cells by enhancing the function of CD8^+^ T cells is expected to be an effective intervention to promote tumor therapy.

**Conclusion:** Our study reveals the pathogenesis and potential biological processes of UV exposure-induced ferroptosis in the development of SKCM, which may provide potential immunotherapy targets for SKCM treatment *via* tumor cell ferroptosis mechanisms.

## Introduction

Skin cutaneous melanoma (SKCM) is the most common malignant tumor of skin cancer with the highest mortality rate. It originates from melanocytes and is highly malignant and prone to metastasis (Guo et al., 2022). Melanocytes produce and release melanin, which acts as a barrier to ultraviolet (UV) radiation, thus preventing UV-induced DNA mutations. However, excessive UV radiation is a risk factor for the development of several skin tumors and solar diseases, and is a major environmental trigger for melanoma. The increased risk of melanoma due to solar exposure is directly related to UV levels (Cabrera and Recule, 2018). Previous studies have shown an association between Sun exposure and melanoma risk, with patients with intense, intermittent Sun exposure and a history of typical sunburn having a higher risk of developing melanoma compared to chronic continuous Sun exposure (Sample and He, 2018). Rastrelli et al. (Rastrelli et al., 2014) found that a history of sunburn in childhood or adolescence was associated with a higher risk of melanoma and that people who experienced more than 5 severe sunburns had a twice the risk of melanoma. Therefore, exposure to the Sun is an important risk factor for the development of melanoma.

The UV of sunlight can cause an excessive intracellular production of reactive oxygen species (ROS), resulting in damage by oxidative stress to cells (Davies, 1995; Rhee, 2006). Mitochondria are the producers and targets of ROS and play an important role in maintaining cellular homeostasis (Annesley and Fisher, 2019). Interesting, as a major iron-rich and ROS-producing organelle, mitochondria are considered as an important place for cell ferroptosis (Gao et al., 2019). As a novel form of programmed cell death, there is increasing evidence that ferroptosis is closely associated with the occurrence and development of malignant tumors (Hirschhorn and Stockwell, 2019; Mou et al., 2019). Activating or inhibiting ferroptosis can effectively affect the activity of tumor cells. Therefore, ferroptosis has good prospects for application in the early diagnosis and treatment of tumors. However, there are no relevant studies to explore the potential mechanism of ferroptosis caused by UV-induced oxidative damage to mitochondria in the onset and development of primary SKCM.

Exploration of cotranscriptional signatures help to understand important links between cell injury and disease. In this study, intrinsic associations with ferroptosis-related genes in a melanoma cell lines datasets (GSE31909) and UV-irradiated mitochondria dataset (GSE3632) were analyzed. The physiological mechanisms of ferroptosis-related DEGs and their important role in the expression, pathological stage, and prognosis of SKCM were explored through a comprehensive bioinformatics analysis. Subsequently, the connection between ferroptosis and tumor immune microenvironment of primary SKCM was explored to provide new therapeutic strategies for ferroptosis-based tumor immunotherapy.

## Materials and methods

### Data collection

Gene expression profiles on melanoma cell lines and UV-irradiated mitochondria were downloaded from GEO database (https://www.ncbi.nlm.nih.gov/geo) (Xiong et al., 2022). The melanoma cell lines datasets (GSE31909) and UV-irradiated mitochondria dataset (GSE3632) were download from GEO for further analysis. The melanoma cell lines datasets were based on the GPL10558 platform (Illumina HumanHT-12 V4.0 expression beadchip), which include samples from 6 normal melanocytes and 6 melanoma samples (3 primary melanoma samples and 3 melanoma samples with lymph node metastasis). The UV-irradiated mitochondria dataset was based on the GPL2570 platform (MBPL Human 30k P4), which includes 4 controls samples and 6 UV-irradiation samples. The details of datasets are shown in Table 1. In this study, the expression data in primary melanoma samples and healthy controls were downloaded and the ferroptosis-related genes were obtained from the FerrDb v2 database (http://www.zhounan.org/ferrdb/current/).

**TABLE 1:** Data collection.

Condition	GEO dataset	Platform	Number of samples
Melanoma	GSE31909	GPL10558	6 normal melanocytes and 6 melanoma samples
Mitochondria Respiratory Chain	GSE3632	GPL2570	4 controls samples and 6 UV irradiation samples

### Differential analysis of gene expression profiles

The gene expression profile data were read by GEOquery packages, and the differential expression fold change was calculated by Limma R packages. The probe without corresponding genes was deleted, and multiple probe sets with the same gene were averaged. For the mRNA expression data set of the melanoma cell line, the threshold of DEGs was set to a *p*-value < 0.05, and a base-2 logarithm with a fold change value greater than ±1. For cDNA expression data set of UV-irradiated mitochondria, the threshold was set to a *p*-value < 0.05. The common DEGs were screened by an online Venn diagram tool.

### Enrichment analysis of differentially expressed genes

DAVID database (https://david.ncifcrf.gov/) is currently the most widely used biological annotation database, enabling rapid enrichment annotation of pathways in the biology of genes. GO annotation enrichment analysis, including molecular function (MF), biological process (BP), and cell component (CC), and KEGG pathway enrichment analysis was performed using DAVID. The enrichment analysis based on the expression of each gene was analyzed and visualized by the “clusterProfiler” R package. In addition, the gene-disease enrichment analysis was performed by using Enrichr database (https://maayanlab.cloud/Enrichr/).

### Co-expression network of differentially expressed genes

The STRING database (https://cn.string-db.org/) was used to search for the regulatory network of protein-protein interactions (PPI), and the GeneMANIA (http://genemania.org/) online database was used to explore the coexpression network. The connectivity degree of each gene in PPI network is calculated using the degree algorithm in cytohubba plug-in, and the gene with the highest node degree is defined as the hub gene. The top 10 hub genes PPI network was visualized with Cytoscape (version 3.7.2) software.

### Expression and survival analysis

The GEPIA database includes tumor and normal sample data from TCGA and GTEx, was used to explore the association between gene expression in coexpression networks in melanoma patients, tumor pathological stage, and overall survival of the patient (OS) and disease-free survival (DFS) (Xiong et al., 2020). The Human Protein Atlas (HPA) database contains many immunohistochemical data of tissues, and the immunohistochemical data of normal skin and SKCM tissues for this study were derived from the HPA public database, so human ethics approval was not needed. Therefore, we revalidated genes with different expression in TCGA on the tissue immunohistochemical specimen.

### Construction of a diagnostic model

To investigate whether the genes can be used to predict the occurrence of SKCM, the expression data of SKCM specimens in TCGA database was extracted using “ggplot” R package. Then, a least absolute shrinkage and selection operator (LASSO) logistic regression model, Cox regression prediction model and ROC analysis prediction model were established by “glmnet” package, “pROC” package and “survival” package, respectively, and used to verify genes effectiveness.

### Immune cell infiltration analysis

The tumor immune microenvironment is an important factor in the onset and development of tumors. CIBERSORTx analytical tool calculate the enrichment status of 22 immune cells based on the gene expression profile of the specimen, LM22 (22 immune cell types) and 1000 permutations as selection parameters. The TIMER (version 2.0) database was used to explore the correlations of hub gene expression with six types of immune cell infiltration abundance, including CD4^+^ T cells, CD8^+^ T cells, B cells, neutrophils, macrophages, and dendritic cells, and the correlation of endothelial cell infiltration abundance and cancer-associated fibroblasts.

### Statistical analysis

All data of two sets comparisons were standardization and analyzed by Z-score algorithm and Student’s t-test, respectively. In this study, A *p*-value < 0.05 was considered significant of comparison of two groups.

## Results

### Identification of differentially expressed genes

**FIGURE 1:**
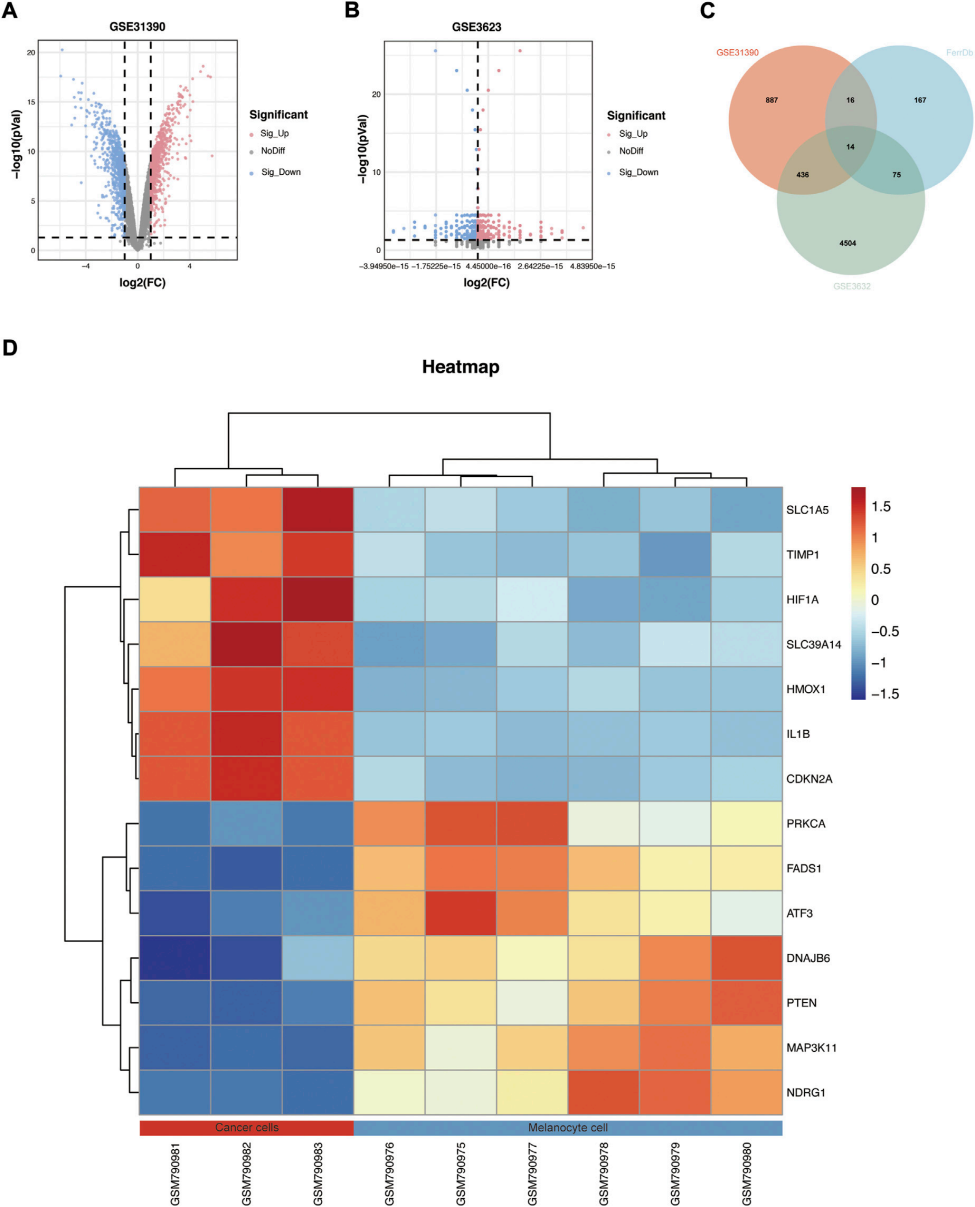
Identification of DEGs. **(A,B)** Volcano map of GSE31909 and GSE3632. **(C)** Venn diagram showed the intersection of common DEGs from the two datasets and ferroptosis-related genes. **(D)** Heatmap showed all common DEGs in GSE31909.

A total of 271 ferroptosis-related genes were downloaded from FerrDb v2 database. The gene expression profiles data were standardized, DEGs (5028 in GSE3632 and 1352 in GSE31909) were identified (Figures 1A,B). After the Venn diagram intersection screening, 14 common DEGs were identified (Figure 1C) and visualized using a heat map (Figure 1D). The detailed descriptions of the common DEGs are listed in Table 2.

**TABLE 2:** The details of the common DEGs.

No.	Gene symbol	Full name	Function
1	IL1B	Interleukin 1 beta	This cytokine is an important mediator of the inflammatory response, and is involved in a variety of cellular activities, including cell proliferation, differentiation, and apoptosis.
2	HMOX1	Heme oxygenase 1	Heme oxygenase, an essential enzyme in heme catabolism.
3	FADS1	Fatty acid desaturase 1	The protein encoded by this gene is a member of the fatty acid desaturase (FADS) gene family. Desaturase enzymes regulate unsaturation of fatty acids through the introduction of double bonds between defined carbons of the fatty acyl chain.
4	CDKN2A	Cyclin dependent kinase inhibitor 2A	This gene is frequently mutated or deleted in a wide variety of tumors, and is known to be an important tumor suppressor gene.
5	MAP3K11	Mitogen-activated protein kinase kinase kinase 11	This kinase can directly phosphorylate, and activates IkappaB kinase alpha and beta, and is found to be involved in the transcription activity of NF-kappaB mediated by Rho family GTPases and CDC42.
6	DNAJB6	DnaJ heat shock protein family (Hsp40) member B6	This gene encodes a member of the DNAJ protein family. DNAJ family members are characterized by a highly conserved amino acid stretch called the “J-domain” and function as one of the two major classes of molecular chaperones involved in a wide range of cellular events, such as protein folding and oligomeric protein complex assembly.
7	NDRG1	N-myc downstream regulated 1	The protein encoded by this gene is a cytoplasmic protein involved in stress responses, hormone responses, cell growth, and differentiation. The encoded protein is necessary for p53-mediated caspase activation and apoptosis.
8	SLC1A5	Solute carrier family 1 member 5	The SLC1A5 gene encodes a sodium-dependent neutral amino acid transporter that can act as a receptor for RD114/type D retrovirus
9	PTEN	Phosphatase and tensin homolog	This gene was identified as a tumor suppressor that is mutated in a large number of cancers at high frequency. It negatively regulates intracellular levels of phosphatidylinositol-3,4,5-trisphosphate in cells and functions as a tumor suppressor by negatively regulating AKT/PKB signaling pathway.
10	TIMP1	TIMP metallopeptidase inhibitor 1	This gene belongs to the TIMP gene family. The encoded protein is able to promote cell proliferation in a wide range of cell types, and may also have an anti-apoptotic function.
11	HIF1A	Hypoxia inducible factor 1 subunit alpha	This gene encodes the alpha subunit of transcription factor hypoxia-inducible factor-1 (HIF-1), which is a heterodimer composed of an alpha and a beta subunit. HIF-1 thus plays an essential role in embryonic vascularization, tumor angiogenesis and pathophysiology of ischemic disease.
12	PRKCA	Protein kinase C alpha	Protein kinase C (PKC) is a family of serine- and threonine-specific protein kinases that can be activated by calcium and the second messenger diacylglycerol. This kinase has been reported to play roles in many different cellular processes, such as cell adhesion, cell transformation, cell cycle checkpoint, and cell volume control.
13	ATF3	Activating transcription factor 3	This gene is induced by a variety of signals, including many of those encountered by cancer cells, and is involved in the complex process of cellular stress response.
14	SLC39A14	Solute carrier family 39 member 14	This gene encodes a member of the SLC39A family of divalent metal transporters that mediates the cellular uptake of manganese, zinc, iron, and cadmium. It is an important transporter of nontransferrin-bound iron and a critical regulator of manganese homeostasis.

### Enrichment analysis of common differentially expressed genes

**FIGURE 2:**
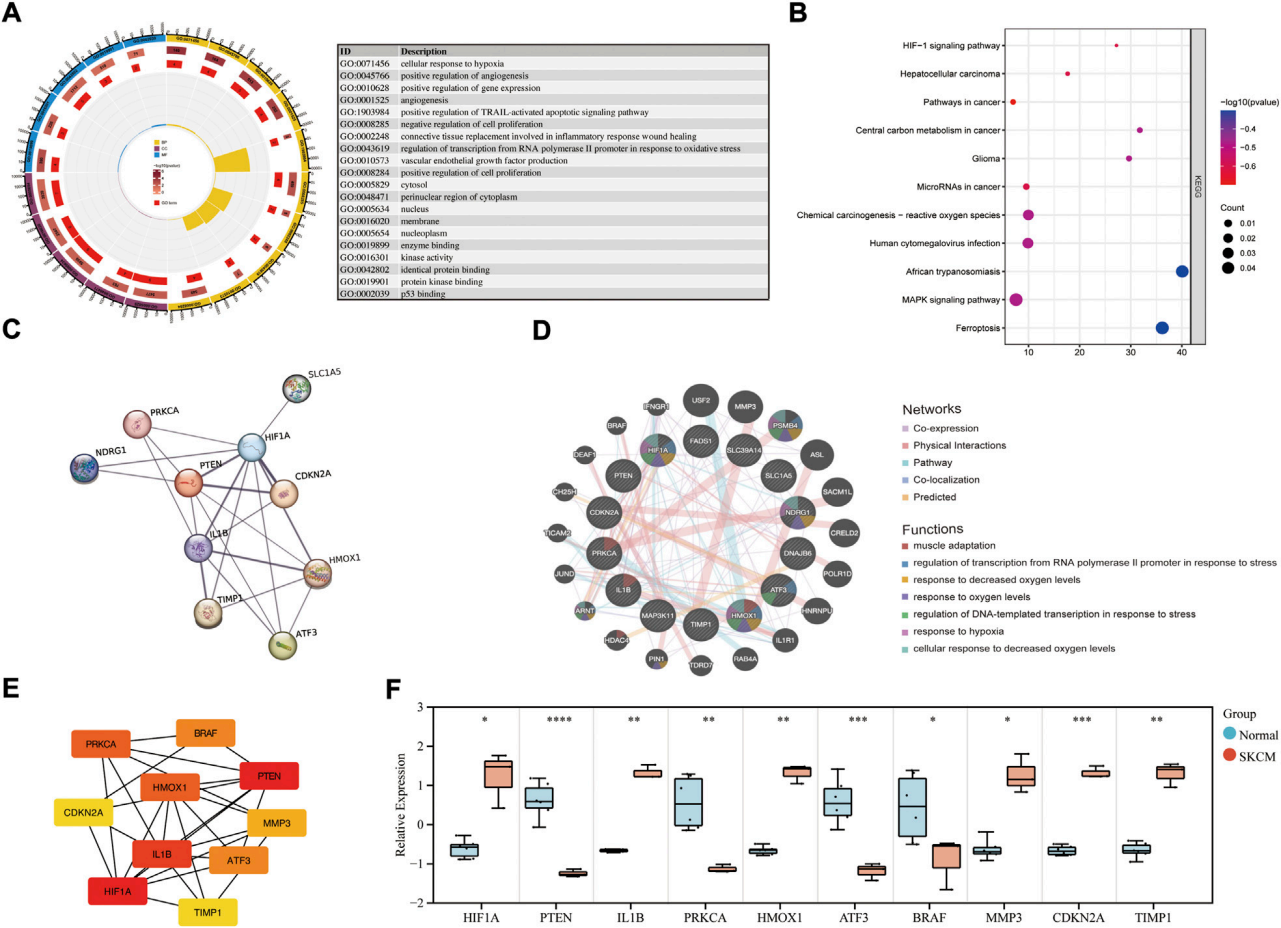
Enrichment analysis of common DEGs and PPI network construction. **(A)** GO enrichment analysis of common DEGs. **(B)** KEGG enrichment analysis of common DEGs. **(C)** PPI network of common DEGs was constructed by STRING. **(D)** Co-expression PPI networks of common DEGs were constructed by GeneMANIA. **(E)** The top 10 hub genes in the co-expression network were obtained and visualized with Cytoscape. **(F)** The mRNA expression level in melanoma cell lines and health controls.

GO annotation, KEGG pathway and gene-disease enrichment analysis were performed to understand the biological functions and pathways of common DEGs. For the enrichment analysis of the GO annotation (Figure 2A), these genes were involved mainly in the cellular response to hypoxia (*p* = 1.02E-04), positive regulation of angiogenesis (*p* = 1.63E-04), cytosol (*p* = 8.78E-03) and the binding of enzymes (*p* = 2.17E-03). For the analysis of the enrichment of the KEGG pathway (Figure 2B), these genes were mainly enriched in the HIF-1 signaling pathway (*p* = 2.60E-04), hepatocellular carcinoma (*p* = 9.26E-04) and cancer pathways (*p* = 2.72E-03). In terms of gene-disease enrichment analysis (Table 3), these genes were mainly enriched in oropharyngeal neoplasms (*p* = 3.74E-07), stage 0 skin melanoma (*p* = 5.34E-07) and secondary malignant neoplasm of prostate (*p* = 4.19E-05). These results indicate that these common DEGs are strongly involved in the onset and development of melanoma.

**TABLE 3:** The top 10 gene-disease enrichment term based on the combined score.

No.	Term	*p*-value	Combined score
1	Oropharyngeal Neoplasms	3.74E-07	4.76E+03
2	Stage 0 Skin Melanoma	5.34E-07	3.99E+03
3	Secondary malignant neoplasm of prostate	4.19E-05	3.14E+03
4	Granuloma Annulare	4.19E-05	3.14E+03
5	Numerous nevi	4.19E-05	3.14E+03
6	Gemistocytic astrocytoma	8.83E-11	2.67E+03
7	Acral Lentiginous Malignant Melanoma	5.86E-05	2.43E+03
8	Chemical Burns	5.86E-05	2.43E+03
9	Conjunctivochalasis	5.86E-05	2.43E+03
10	Pigmented lesions	1.61E-06	2.34E+03

### Protein-protein interactions network construction and analysis

The PPI network of the common DEGs was constructed and analyzed using the STRING database (Figure 2C). Then, the coexpression PPI networks of the common DEGs were constructed by the GeneMANIA database (Figure 2D). The co-expression PPI networks with the co-expression (41.76%), physical interactions (40.18%), co-localization (3.65%) and predicted (3.11%). The top seven gene functions were displayed in the coexpression expression network, muscle adaptation (FDR = 8.03E-03, Coverage = 4/53), regulation of transcription from RNA polymerase ΙΙ promoter response to stress (FDR = 8.03E-03, Coverage = 5/104) and response to decreased oxygen levels (FDR = 8.03E-03, Coverage = 6/208) were the most enrichment function pathway. Then, the degree algorithm calculates the linkage connectivity of each gene in the co-expression network, and the top 10 hub genes are visualized with Cytoscape (Figure 2E). The relative expression level of the hub genes was reverified in melanoma cell lines (Figure 2F), and showed that HIF1A, IL1B, HMOX1, MMP3, CDKN2A and TIMP1 were upregulated, while PTEN, PRKCA, ATF3 and BRAF were downregulated in melanoma samples.

**FIGURE 3:**
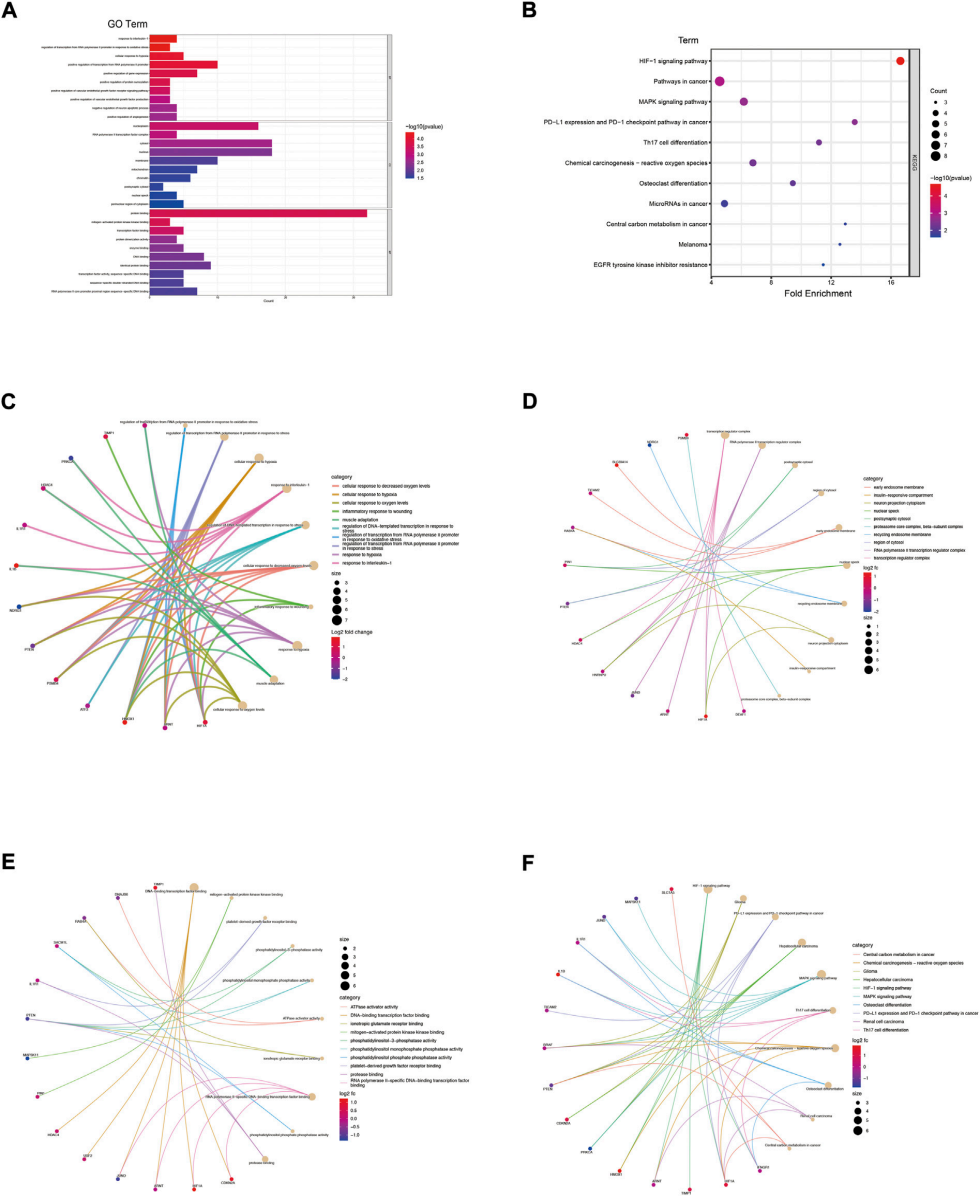
Enrichment analysis of genes in co-expression PPI networks. **(A)** GO enrichment analysis of these genes. **(B)** KEGG enrichment analysis of these genes. **(C)** The category of “biological process” of these genes was displayed by chord diagram. **(D)** The category of “cellular component” of these genes was displayed by chord diagram. **(E)** The category of “molecular function” of these genes was displayed by chord diagram. **(F)** The KEGG pathway enrichment analysis of these genes was displayed by chord diagram.

The genes in the co-expression network were also performed to enrichment analysis. In terms of BP (Figures 3A,C; Supplementary Table S1), the genes were mainly involved in response to interleukin-1 (*p* = 3.95E-05), regulation of transcription of the RNA polymerase II promoter in response to oxidative stress (*p* = 4.00E-05) and cellular response to hypoxia (*p* = 8.22E-05). In terms of CC (Figures 3A,D; Supplementary Table S2), the genes were components of the nucleoplasm (*p* = 5.42E-04), RNA polymerase II transcription factor complex (*p* = 9.65E-04) and the cytosol (*p* = 2.12E-03). In term of CC (Figures 3A,E; Supplementary Table S3), the genes were mainly involved in protein binding (*p* = 2.28E-04), mitogen-activated protein kinase kinase binding (*p* = 2.30E-04) and transcription factor binding (*p* = 4.71E-04). The KEGG enrichment analysis result (Figures 3B,F) showed that HIF-1 signaling pathway (*p* = 2.04E-05) and pathways in cancer (*p* = 1.06E-03) were the most enrichment pathway.

### Expression, tumor pathological stage and survival analysis

**FIGURE 4:**
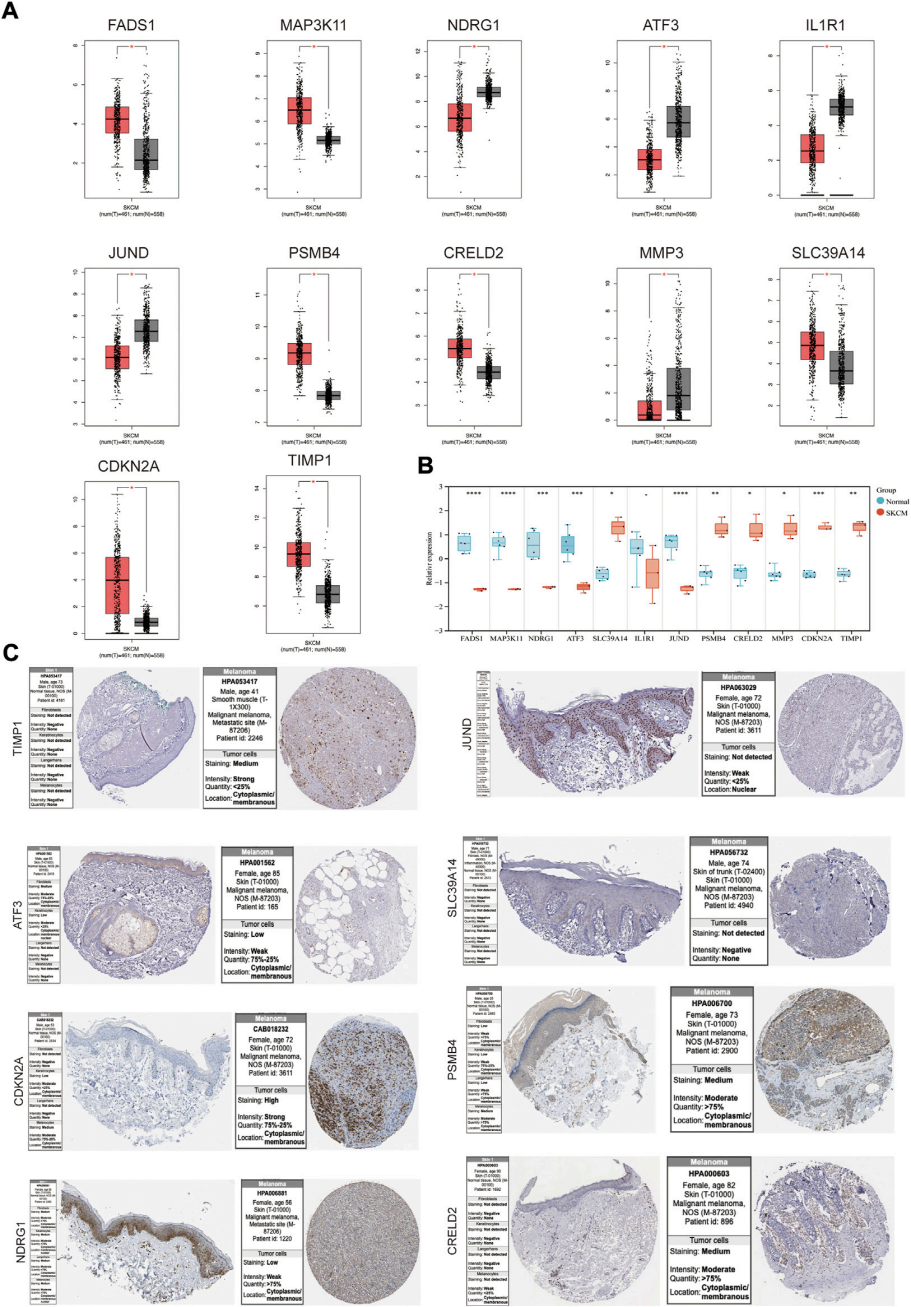
The mRNA and protein expression levels of genes in co-expression networks with melanoma patients. **(A)** Gene expression profile of genes in co-expression networks in GEPIA. **(B)** Gene expression profile of these genes in melanoma cell lines and health controls. **(C)** The protein expression levels of the genes in HPA. *p < 0.05; **p < 0.01; ***p < 0.001; ****p < 0.0001.

The mRNA expression levels of genes in the coexpression network were explored using GEPIA. FADS1, MAP3K11, SLC39A14, PSMB4, CRELD2, CDKN2A and TIMP1 were higher in SKCM tissues than normal skin tissues, and NDRG1, ATF3, IL1R1, JUND and MMP3 had an opposite trend (Figure 4A). The expression patterns of these genes were then verified in melanoma cell lines (Figure 4B), and SLC39A14, PSMB4, CRELD2, CDKN2A, TIMP1, NDRG1, ATF3, and JUND were consistent with the above trends. Protein expression levels of these genes were evaluated in normal skin and SKCM tissues (Figure 4C). The results showed that except for SLC39A14, the protein expression of the other seven genes was consistent with the expression of mRNA in the SKCM samples. In particular, CDKN2 A, PSMB4, and CRELD2 were significantly enhanced compared to normal skin immunohistochemical staining.

**FIGURE 5:**
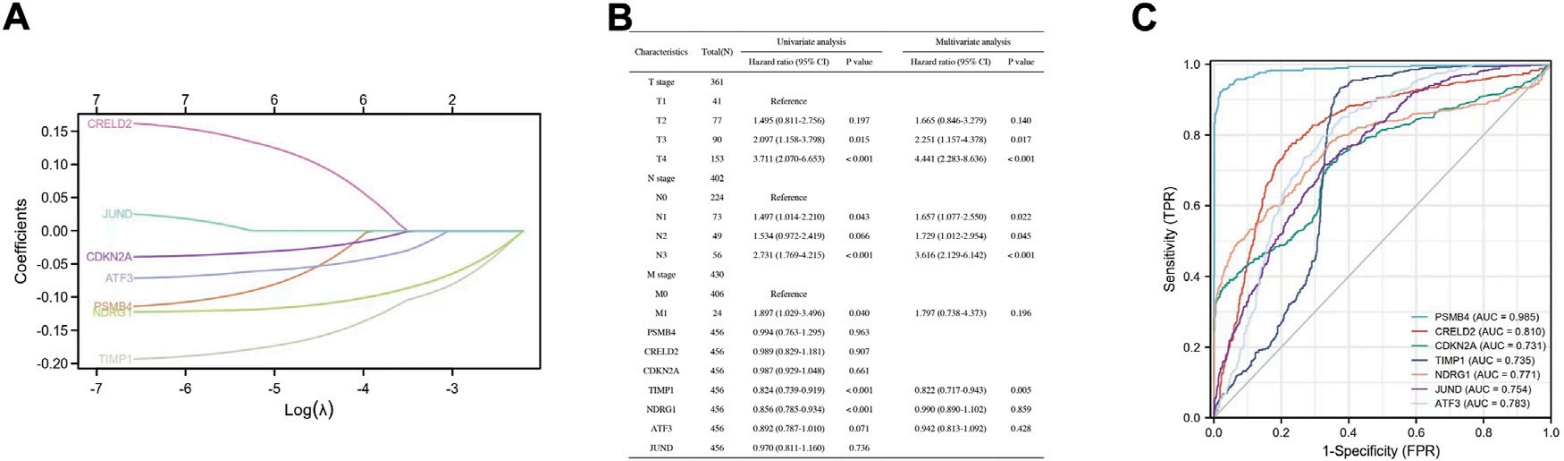
Construction of a diagnostic model to verify the DEGs effectiveness of SKCM prediction. **(A)** LASSO logistic regression algorithm to verify the DEGs. **(B)** Univariate and multivariate analysis of the DEGs using the Cox proportional hazard regression model. **(C)** The ROC curve of the diagnostic efficacy verification.

To investigate whether these DEGs (PSMB4, CRELD2, CDKN2A, TIMP1, NDRG1, ATF3, and JUND) can be used to predict the appearance of SKCM, the expression profile of these DEGs from the TCGA database was extracted to construct the LASSO model, and it was found that the genes can be identified with nonzero regression coefficients (Figure 5A). Univariate and multivariate analysis of these DEGs using the Cox proportional hazard regression model (Figure 5B). For univariate analysis results, TIMP1 (Hazard ratio = 0.824, 95% CI = 0.739–0.919, *p* < 0.001) and NDRG1 (Hazard ratio = 0.856, 95% CI = 0.785–0.934, *p* < 0.001) have significant correlation with SKCM. For the results of the multivariate analysis, TIMP1 (Hazard ratio = 0.822, 95% CI = 0.717–0.943, *p* = 0.005) has a significant correlation with SKCM. Based on the TCGA database, ROC analysis was used to validate the diagnostic effectiveness of these DEGs for SKCM (Figure 5C). The value of area under curve (AUC) for these genes were all greater than 0.7, and PSMB4 (AUC = 0.985) and CRELD2 (AUC = 0.81) were the most diagnostic.

**FIGURE 6:**
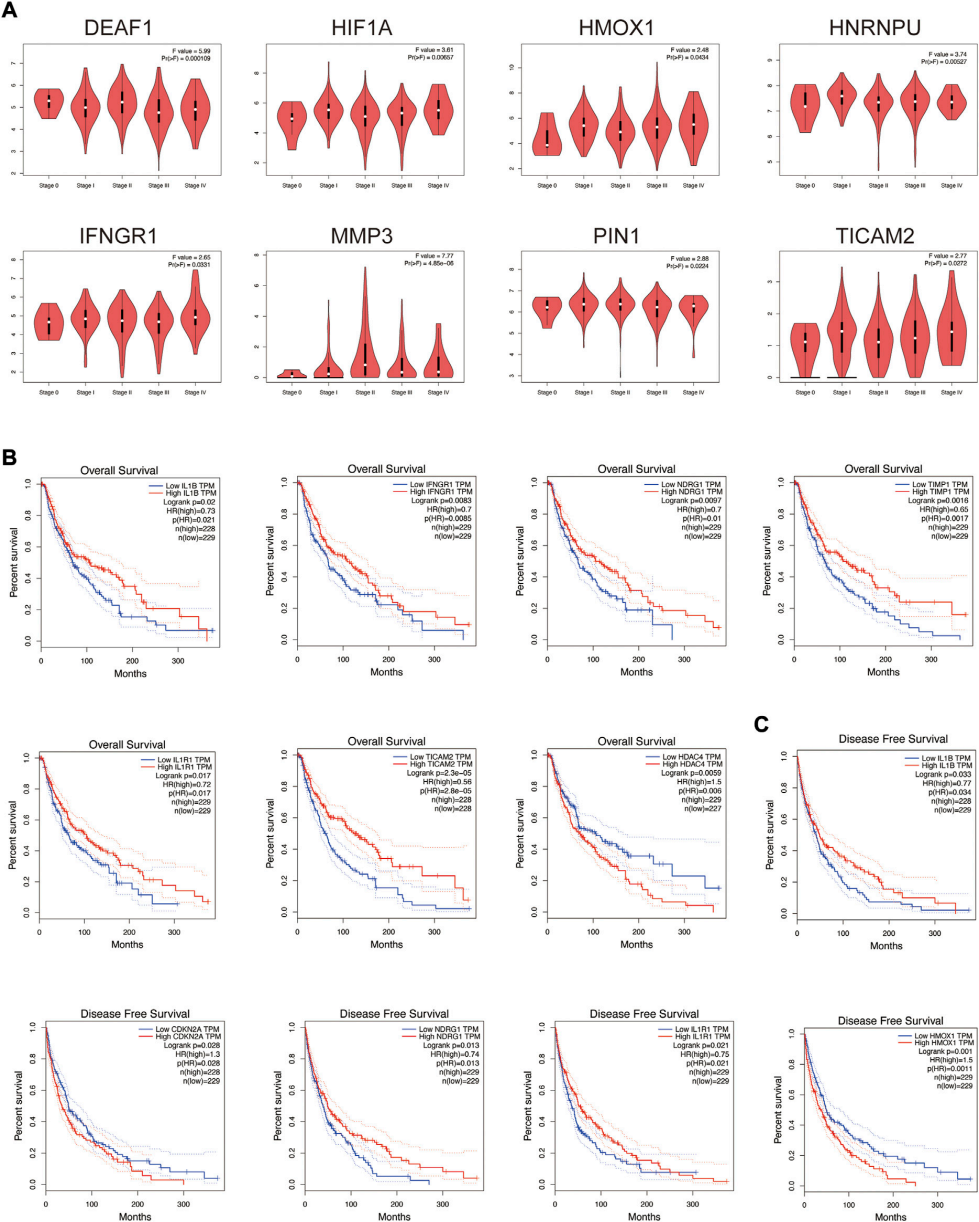
Tumor pathological stage and multifaceted prognostic value of co-expression genes in SKCM. **(A)** Correlation between these genes’ expression and tumor stage in SKCM patients with GEPIA. **(B)** The overall survival prognostic value of mRNA level of these genes in SKCM patients. **(C)** The disease-free survival prognostic value of mRNA level of these genes in SKCM patients.

The relationship between these co-expression genes and tumor pathological stage and multifaceted prognostic value were explored. For tumor pathological stage (Figure 6A), the results indicated that MMP3, DEAF1, IFNGR1, HIF1A, PIN1, HNRNPU, TICAM2 and HMOX1 groups varied significantly (*p* < 0.05), and MMP3 was highly correlated with stage II skin melanoma (F value = 7.77, *p* = 4.85E-06). For the multifaceted prognostic value index (OS and DFS) in patients with SKCM (Figures 6B,C), the results suggested that the mRNA levels of IL1B, IFNGR1, NDRG1, TIMP1, IL1R1, TICAM2 and HDAC4 were significantly associated with OS in patients with SKCM as detrimental prognostic factors (IL1B logrank *p* = 0.02, IFNGR1 logrank *p* = 0.0083, NDRG1 logrank *p* = 0.0097, TIMP1 logrank *p* = 0.0016, IL1R1 logrank *p* = 0.017, TICAM2 logrank *p* = 2.30E-05, HDAC4 logrank *p* = 0.0059), and IL1B, CDKN2A, NDRG1, IL1R1 and HMOX1 mRNA levels were significantly associated with DFS (IL1B logrank *p* = 0.033, CDKN2A logrank *p* = 0.028, NDRG1 logrank *p* = 0.013, IL1R1 logrank *p* = 0.021, HMOX1 logrank *p* = 0.001). Patients with low IL1B, NDRG1, and IL1R1 mRNA levels were significantly associated with low OS and DFS.

### Association of hub genes expression with immune cell infiltration in skin cutaneous melanoma

**FIGURE 7:**
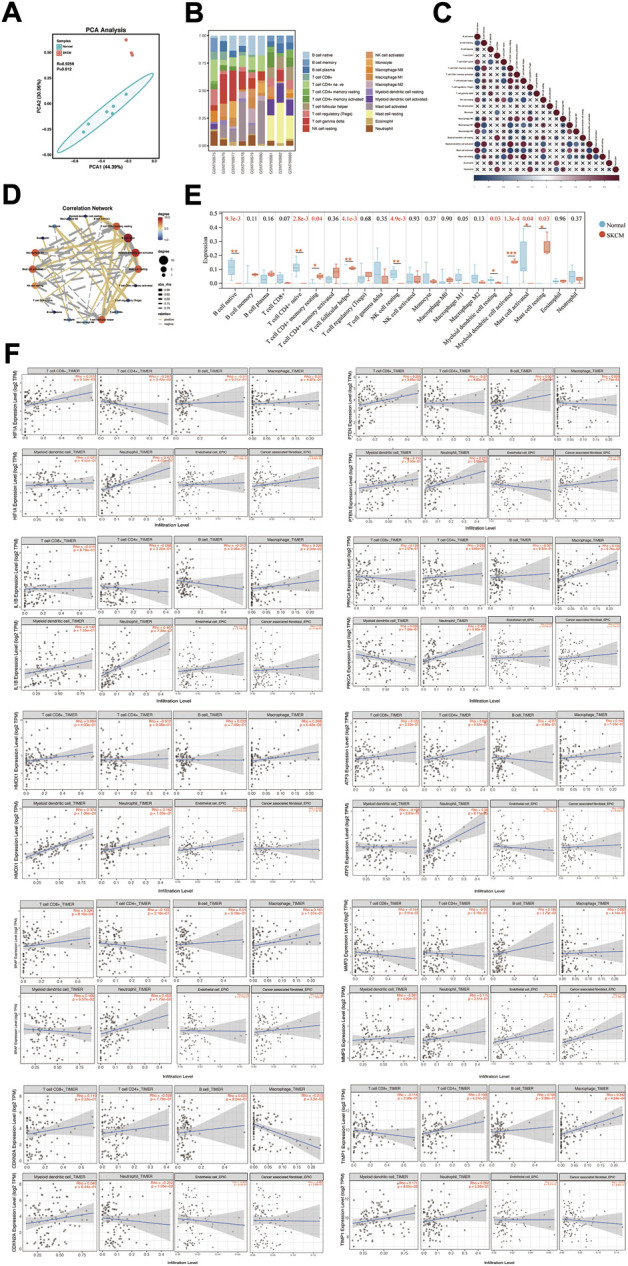
Immune cell infiltration status in SKCM. **(A)** PCA cluster plot of immune cell infiltration between melanoma cell lines and health controls. **(B)** Histogram showed the composition of 22 kinds of immune cells in both samples. **(C)** Correlation heat map of 22 types of immune cells. The size of the colored squares represents the strength of the correlation; blue represents negative correlation, red represents a positive correlation. “×” represents *p* value > 0.05. **(D)** Correlation network map showed a connected network with immune cells. *p* value < 0.05, and Rho ≥ 0.5 or Rho ≤ −0.5 were showed in the network map. **(E)** Box diagram of the infiltration of immune cells between the two groups of samples. *p < 0.05; **p < 0.01; ***p < 0.001. **(F)** Correlation between different expressed hub genes and immune cell infiltration in TIMER.

Tumor microenvironment is an important factor contributing to the progression of tumorigenesis. Infiltration of 22 types of immune cells was analyzed in melanoma cell lines and normal controls using the CIBERSORT algorithm, and the results of the PCA grouping analysis showed significant differences (R = 0.9295, *p* = 0.012) in the enrichment of immune cells between the two samples (Figure 7A). Figure 7B shows the proportion of immune cells in 22 in each sample. Correction analysis of immune cells in melanoma samples revealed that native B cells had a significant positive correlation with memory resting CD4^+^ T cells, native CD 4^+^ T cells, M1 macrophages, M2 macrophages, activated mast cells, resting NK cells, and dendritic cells (Figures 7C,D). We found that melanoma samples had higher levels of infiltration of memory resting CD4^+^ T cell, helper follicular T cell, activated dendritic cell and resting mast cell, and had lower levels of infiltration of native B cell, native CD 4^+^ T cell, resting NK cell, resting dendritic cell, and activated mast cell (Figure 7E).

Subsequently, the association of the expression of hub genes with the infiltration of immune cells from the tumor microenvironment in SKCM was analyzed by the TIMER database (Figure 7F). HIF1A positively correlates with CD 8^+^ T cell infiltration (Rho = 0.255, *p* = 9.59e-03), and negatively correlates with CD 4^+^ T cell (Rho = −0.287, *p* = 3.42e-03) and neutrophils (Rho = −0.477, *p* = 4.05e-07) infiltration. PTEN is positively correlated with infiltration of CD 8^+^ T cells (Rho = 0.205, *p* = 3.85e-02) and neutrophils (Rho = 0.272, *p* = 5.69e-03) infiltration. IL1B positively correlates with macrophages (Rho = 0.229, *p* = 2.04e-02), neutrophils (Rho = 0.467, *p* = 7.38e-07) and endothelial cells (Rho = 0.212, *p* = 3.19e-02) infiltration. PRKCA is positively correlated with neutrophils (Rho = 0.468, *p* = 6.92e-07). HMOX1 positively correlates with macrophages (Rho = 0.268, *p* = 6.42e-03) and dendritic cells (Rho = 0.374, *p* = 1.06e-04) infiltration. ATF3 is positively correlated with neutrophil infiltration (Rho = 0.38, *p* = 8.11e-05) infiltration. BRAF is positively correlated with CD 8^+^ T cell infiltration (Rho = 0.326, *p* = 8.16e-04) and neutrophils (Rho = 0.453, *p* = 1.79e-06) infiltration. MMP3 is positively correlated with endothelial cell infiltration (Rho = 0.285, *p* = 3.48e-03) and cancer-associated fibroblasts (Rho = 0.242, *p* = 1.39e-02) infiltration. CDKN2A is negatively correlated with macrophage infiltration (Rho = −0.213, *p* = 3.2e-02) and neutrophils (Rho = −0.252, *p* = 1.05e-02) infiltration. TIMP1 is positively correlated with CD 4^+^ T cell (Rho = 0.199, *p* = 4.51e-02) and macrophage (Rho = 0.342, *p* = 4.34e-04) infiltration.

## Discussion

SKCM is a highly malignant and insidious type of skin tumors, and its incidence is increasing rapidly. Currently, it is the leading tumor type for skin cancer mortality (Abbas et al., 2014). Surgical resection of primary and metastatic lesions, chemotherapy, immunotherapy, radiation therapy, and targeted therapy have long been the mainstay of treatment for SKCM (Kaštelan et al., 2018). However, the effectiveness of these treatments is gradually decreasing due to the presence of multiple resistance mechanisms, and new therapeutic strategies are urgently needed.

Various biological therapies such as cellular immunotherapy, cytokine, and monoclonal antibody therapies have been widely used in the treatment of SKCM, and in-depth research on the pathogenesis of SKCM and the discovery of new therapeutic targets is still the current focus (Wong et al., 2017). Numerous studies have confirmed that sunlight exposure is an important environmental factor in the pathogenesis of SKCM (Kopf et al., 1984; Elwood, 1996; Oliveria et al., 2006). Sunlight-driven alterations in DNA chemistry and DNA replication errors are potential mechanisms driving melanocyte mutations (Jin et al., 2021). Mitochondria, an ubiquitous organelle in eukaryotic cells, have a much higher rate of DNA mutation than nuclear DNA and may be involved in the occurrence and drug resistance of SKCM. Wu et al. (2019) found that mitochondrial DNA stress in TFAM-deficient mice produced more drug-resistant melanomas. Thus, the mitochondria is an important organelle in the biological process of melanoma.

Ferroptosis has recently been identified as a natural tumor suppressor mechanism, and mitochondria are important organelles regulating ferroptosis. The pathology and potential biological process of UV-exposure-induced mitochondrial ferroptosis in the development of SKCM have aroused our strong interest. In this study, we explored the cotranscriptional signatures of UV exposure-induced mitochondria and ferroptosis to reveal the intrinsic mechanisms of SKCM. Based on the enrichment results of the common DEGs, these genes are mainly enriched in cellular response to hypoxia, positive regulation of angiogenesis, and the HIF-1 signaling pathway. Hypoxia and intratumoral angiogenesis are common features of solid tumors and contribute to cancer progression and drug resistance. The process of neoangiogenesis has been shown to be critical for melanoma proliferation and is believed to be so primarily in the metastatic process (Dutcher, 2001; Mahabeleshwar and Byzova, 2007). In particular, Dratkiewicz et al. (2021) found that hypoxia reprograms melanoma cell metabolism, enhances tumor cell survival and invasion, and promotes an immunosuppressive environment. Furthermore, hypoxia and vascularization are mutually strengthening (Malekan et al., 2021) Numerous studies have shown that upregulation of HIF-1α/VEGF signaling pathway will promote tumor cell migration, invasion and melanoma angiogenesis (Zheng et al., 2020; Roy et al., 2021; Xu et al., 2021). These results showed that hypoxia and vascularization of the local microenvironment of cancerous cells caused by mitochondrial ferroptosis induced by ultraviolet irradiation may be an important factor in the occurrence and development of SKCM.

PPI coexpression networks contribute to increasing understanding of functional connections between proteins under disease conditions. We obtained the top 10 genes with the most nodal degree in the PPI coexpression network by using the degree algorithm. HIF1A, PTEN, and IL1B were found to be the main hub gene for the coexpression network. The high expression of HIFA in melanoma samples is consistent with the characteristics of hypoxia and angiogenesis of SKCM tissue (Figure 2F). PTEN, a tumor suppressor, whose down-regulation would cause the development of SKCM (Dankort et al., 2009). Lin et al. (2021) found that PTEN deficiency was associated with adverse responses and resistance to the immunosuppressive tumor microenvironment, and combined PTEN mRNA-containing nanoparticles with PD-1 antibodies exhibited efficient antitumor effects. Interestingly, genes in coexpression networks such as HIF1A, PTEN, and IFNGR1 were enriched in the expression of PD-L1 and the PD-1 checkpoint pathway in cancer (Figures 3B,F). Pro-inflammatory cytokines can regulate the proliferation and survival of cutaneous melanoma cells. IL1B is an important mediator of the inflammatory response, higher levels of melanoma cells were associated with proliferation, invasion, and migration of melanoma cells (Torricelli et al., 2021). The expression pattern of the hub genes is an important factor in the occurrence and progression of SKCM.

Understanding the expression pattern of genes and proteins in tissues with melanoma can effectively improve the early diagnosis and prognosis of SKCM. We examined the mRNA and protein expression levels of genes in the co-expression network in SKCM. For the level of mRNA expression in the TCGA database and melanoma cell lines, SLC39A14, PSMB4, CRELD2, CDKN2A and TIMP1 were higher in SKCM tissues than in normal skin tissues, and NDRG1, ATF3, and JUND had an opposite trend. For protein expression level in HPA database, except for SLC39A14, the protein expression of the other seven genes was consistent with the mRNA expression in SKCM samples. Then, to investigate whether these DEGs (PSMB4, CRELD2, CDKN2A, TIMP1, NDRG1, ATF3 and JUND) can be used to predict the occurrence of SKCM, the expression profile of these DEGs from the TCGA database was extracted to construct the LASSO model, Cox proportional risk regression model and ROC analysis prediction model, and found that PSMB4, CRELD2, CDKN2A, TIMP1, NDRG1, ATF3 and JUND have expression consistency and prediction stability, which can be used as a molecular marker for early diagnosis of SKCM (Figures 4, 5). Interestingly, CDKN2A, TIMP1, NDRG1 and JUND are closely associated with HIF-1 pathway. Numerous studies have shown that there was a positive correlation between the expression patterns of TIMP1 and CDKN2A and HIF-1α (Zhang et al., 2019; Kim et al., 2022), and the expressions of NDRG1 and JUND were negatively correlated with HIF-1α (Toullec et al., 2010; Ercin et al., 2019). Therefore, abnormal expression of these genes in SKCM may lead to activation of the HIF-1 signaling pathway, which will promote the vascularization process of SKCM, leading to tumor progression.

Then, the relationship between these co-expression genes and tumor pathological stage and multifaceted prognostic value were explored. MMP3 was highly correlated with stage II skin melanoma (F value = 7.77, *p* = 4.85E-06). Shoshan et al. (2016) found *in vivo* studies that MMP3 was an important factor promoting melanoma tumor growth and lung metastasis. The prognostic correlation analysis of SKCM with molecular markers found that patients with low mRNA levels of IL1B, NDRG1, and IL1R1 were significantly associated with low OS and DFS. Notably, IL1B, NDRG1 and TIMP1 act as drivers of ferroptosis, and their low expression in patients with poor prognosis implies a correlation between tumor cell resistance to ferroptosis mechanisms and poor prognosis. This result indicated that the promotion of tumor cell ferroptosis is expected to be an effective means of improving the prognosis of patients with SKCM.

Growing evidence suggests that tumor immune microenvironment is an important factor affecting tumor progression, and tumor immunotherapy is expected to be an effective means to delay tumor progression (Liu et al., 2017; Guo et al., 2022). Therefore, we explored the enrichment status of immune cells in melanoma samples, and showed that melanoma samples had higher levels of infiltration of memory resting CD4^+^ T cells, helper follicular T cells, activated dendritic cells and resting mast cells, and had lower levels of infiltration of native B cells, native CD4^+^ T cells, resting NK cells, resting dendritic cells and activated mast cells (Figure 7E). Wang et al. (2019) found that CD8^+^ T cells enhanced ferroptosis-specific lipid peroxidation in tumor cells. In this study, we found that a high mRNA level of HIF1A (a ferroptosis suppressor) and a low mRNA level of PTEN (a ferroptosis driver) positively correlate with CD8^+^ T cell infiltration, implying that a ferroptosis resistance mechanism exists in the tumor microenvironment of SKCM, and it is expected that the induction of ferroptosis in tumor cells by enhancing the function of CD8^+^ T cells is expected to be an effective intervention to promote tumor therapy.

## Conclusion

**FIGURE 8:**
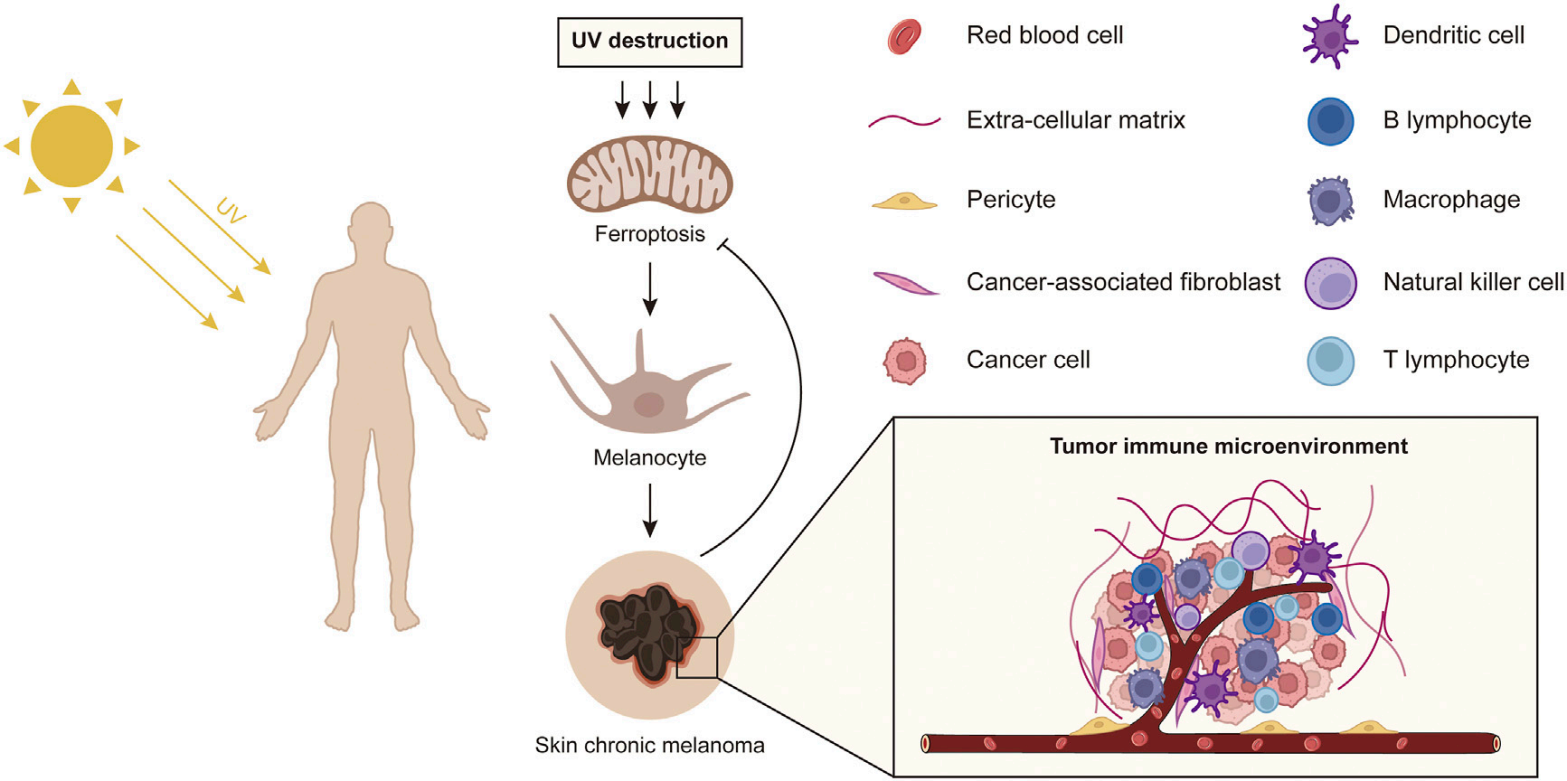
A schematic diagram shows the effect of sun exposure-induced ferroptosis mechanisms on potential biological processes of primary melanoma.

In summary, this study provides a comprehensive analysis of the mechanisms of ferroptosis induced by Sun exposure on the pathology and potential biological processes of primary melanoma and explored the impact of ferroptosis-related genes on the immune microenvironment of the tumor (Figure 8). Our findings help to provide potential immunotherapy targets for SKCM treatment via tumor cell ferroptosis mechanisms.

## Data Availability

The datasets presented in this study can be found in online repositories. The names of the repository/repositories and accession number(s) can be found in the article/Supplementary Material.
