# Supplementary material for: Effect of Sun exposure-induced ferroptosis mechanisms on pathology and potential biological processes of primary melanoma by microarray data analysis
Source: Front Genet. 2022 Sep 26;13:998792. doi: 10.3389/fgene.2022.998792 (PMC9548870; doi:10.3389/fgene.2022.998792)
Supplement: Supplementary file 1 [file Table1.DOCX]

Table 1: The top 10 category of BP in the GO enrichment analysis of the genes in co-expression PPI networks.

| No. | Term ID | Term Name | Count | p-value | Fold Enrichment |
| --- | --- | --- | --- | --- | --- |
| 1 | GO:0070555 | response to interleukin-1 | 4 | 3.95E-05 | 58.35 |
| 2 | GO:0043619 | regulation of transcription from RNA polymerase II promoter in response to oxidative stress | 3 | 4.00E-05 | 291.76 |
| 3 | GO:0071456 | cellular response to hypoxia | 5 | 8.22E-05 | 20.84 |
| 4 | GO:0045944 | positive regulation of transcription from RNA polymerase II promoter | 10 | 1.05E-04 | 4.86 |
| 5 | GO:0010628 | positive regulation of gene expression | 7 | 1.74E-04 | 7.96 |
| 6 | GO:0033235 | positive regulation of protein sumoylation | 3 | 2.06E-04 | 134.66 |
| 7 | GO:0030949 | positive regulation of vascular endothelial growth factor receptor signaling pathway | 3 | 4.03E-04 | 97.25 |
| 8 | GO:0010575 | positive regulation of vascular endothelial growth factor production | 3 | 1.13E-03 | 58.35 |
| 9 | GO:0043524 | negative regulation of neuron apoptotic process | 4 | 2.26E-03 | 14.77 |
| 10 | GO:0045766 | positive regulation of angiogenesis | 4 | 2.34E+03 | 14.23 |

Table 2: The top 10 category of CC in the GO enrichment analysis of the genes in co-expression PPI networks.

| No. | Term ID | Term Name | Count | p-value | Fold Enrichment |
| --- | --- | --- | --- | --- | --- |
| 1 | GO:0005654 | nucleoplasm | 16 | 5.42E-04 | 2.45 |
| 2 | GO:0090575 | RNA polymerase II transcription factor complex | 4 | 9.65E-04 | 19.90 |
| 3 | GO:0005829 | cytosol | 18 | 2.12E-03 | 1.98 |
| 4 | GO:0005634 | nucleus | 18 | 5.28E-03 | 1.83 |
| 5 | GO:0016020 | membrane | 10 | 1.67E-02 | 2.36 |
| 6 | GO:0005739 | mitochondrion | 7 | 2.55E-02 | 2.93 |
| 7 | GO:0000785 | chromatin | 6 | 2.59E-02 | 3.42 |
| 8 | GO:0099524 | postsynaptic cytosol | 2 | 2.71E-02 | 70.84 |
| 9 | GO:0016607 | nuclear speck | 4 | 3.19E-02 | 5.58 |
| 10 | GO:0048471 | perinuclear region of cytoplasm | 5 | 3.33E-02 | 3.95 |

Table 3: The top 10 category of MF in the GO enrichment analysis of the genes in co-expression PPI networks.

| No. | Term ID | Term Name | Count | p-value | Fold Enrichment |
| --- | --- | --- | --- | --- | --- |
| 1 | GO:0005515 | protein binding | 32 | 2.28E-04 | 1.41 |
| 2 | GO:0031434 | mitogen-activated protein kinase kinase binding | 3 | 2.30E-04 | 127.68 |
| 3 | GO:0008134 | transcription factor binding | 5 | 4.71E-04 | 13.24 |
| 4 | GO:0046983 | protein dimerization activity | 4 | 3.41E-03 | 12.79 |
| 5 | GO:0019899 | enzyme binding | 5 | 4.63E-03 | 7.09 |
| 6 | GO:0003677 | DNA binding | 8 | 6.09E-03 | 3.44 |
| 7 | GO:0042802 | identical protein binding | 9 | 8.13E-03 | 2.91 |
| 8 | GO:0003700 | transcription factor activity, sequence-specific DNA binding | 5 | 1.55E-02 | 4.99 |
| 9 | GO:1990837 | sequence-specific double-stranded DNA binding | 5 | 1.57E-02 | 4.98 |
| 10 | GO:0000978 | RNA polymerase II core promoter proximal region sequence-specific DNA binding | 7 | 1.71E-02 | 3.21 |
